# Gastrodin ameliorates learning and memory impairment in rats with vascular dementia by promoting autophagy flux via inhibition of the Ca^2+^/CaMKII signal pathway

**DOI:** 10.18632/aging.202667

**Published:** 2021-03-10

**Authors:** Ting-Ting Chen, Xue Zhou, Yi-Ni Xu, Yue Li, Xiao-Ying Wu, Quan Xiang, Ling-Yun Fu, Xiao-Xia Hu, Ling Tao, Xiang-Chun Shen

**Affiliations:** 1 The High Efficacy Application of Natural Medicinal Resources Engineering Center of Guizhou Province and The High Educational Key Laboratory of Guizhou Province for Natural Medicinal Pharmacology and Druggability, School of Pharmaceutical Sciences, Guizhou Medical University, Guiyang 550025, P.R. China; 2 Guiyang Maternal and Child Health-Care Hospital, Guiyang 550000, P.R. China; 3 The Key Laboratory of Optimal Utilization of Natural Medicine Resources and The Union Key Laboratory of Guiyang City, Guizhou Medical University, School of Pharmaceutical Sciences, Guiyang 550025, P.R. China; 4 The Key Laboratory of Endemic and Ethnic Diseases of Ministry of Education, Guizhou Medical University, Guiyang 550025, P.R. China

**Keywords:** gastrodin, vascular dementia, autophagic flux, Ca^2+^, CaMKII

## Abstract

Vascular dementia (VD) is a common disease that occurs during human aging. Gastrodin (GAS) has potential benefits for the prevention and treatment of VD. In the present study, we investigated the effects of GAS on cognitive dysfunction in rats with VD induced by permanent middle cerebral artery occlusion (pMCAO) and explored the underlying mechanism. Immunohistochemical and western blot analyses revealed that GAS attenuated hippocampal levels of LC3 (microtubule-associated protein 1 light chain 3), p62, and phosphorylated CaMKII (Ca^2+^-calmodulin stimulated protein kinase II) in VD rats. Additionally, our results revealed that cobalt chloride blocked autophagic flux in HT22 cells, which was confirmed by increased levels of LC3 and p62 when combined with chloroquine. Notably, GAS ameliorated the impaired autophagic flux. Furthermore, we confirmed that GAS combined with KN93 (a CaMKII inhibitor) or CaMKII knockdown did not impact the reduced p62 levels when compared with GAS treatment alone. Furthermore, a co-immunoprecipitation assay demonstrated that endogenous p62 bound to CaMKII, as confirmed by mass spectrometric analysis after the immunoprecipitation of p62 from HT22 cells. These findings revealed that GAS attenuated autophagic flux dysfunction by inhibiting the Ca^2+^/CaMKII signaling pathway to ameliorate cognitive impairment in VD.

## INTRODUCTION

The incidence of dementia is growing with an increasingly aging population, and vascular dementia (VD) is one of the most common types documented [1]. Currently, patients with VD exhibit severe neurological signs and symptoms, including increased confinement, paralysis, and loss of activities of daily living, which gradually worsen over time [2]. Clinical treatments for VD include cholinesterase inhibitors, calcium channel blockers, and Meijingang. However, the expected therapeutic effects are rarely achieved in patients with VD [3]. Thus, exploring the potential key molecular targets of VD, as well as novel pharmacological mechanisms for the development of specific and efficient therapeutic drugs, may serve as a promising strategy for the prevention and treatment of VD.

VD is an acquired cognitive impairment syndrome characterized by learning and memory disabilities caused by cerebrovascular diseases [4, 5]. Chronic cerebral hypoperfusion leads to insufficient blood and oxygen supply to the brain and disturbance of energy metabolism, resulting in cerebral infarction. Ultimately, neurons are reduced and the material basis for the performance of brain functions is lost [6, 7]. This pathophysiological mechanism is common and leads to a decline in cognitive abilities in VD [8]. Learning and memory functions are an advanced neurophysiological activity of the brain and an important indicator for drug intervention in VD animals based on clinical and experimental research [5]. In the central nervous system, the hippocampus is involved in learning and memory and is extremely sensitive to damage induced by ischemia and hypoxia [9, 10].

Autophagy is a specific pathway to degrade long-lived proteins and damaged organelles. Furthermore, it is an important route for cells to adapt to adverse environments, maintain a steady-state, and promote survival [11]. Under physiological conditions, autophagy plays an important role in maintaining neuronal function and homeostasis, but overactivation of autophagy can lead to nerve-cell death, namely, autophagic cell death [12]. Accumulating evidence has revealed that autophagy is associated with neurodegenerative and cerebrovascular diseases, playing an important role in the pathogenesis of these diseases [13]. Notably, neurodegenerative diseases are caused by the continuous accumulation of LC3-phosphatidylethanolamine conjugate (LC3-II), the inability of autophagosomes to combine with lysosomes [14], and impairment of autophagic-lysosomal degradation in neurons [15]. Consequently, numerous abnormally aggregated proteins are formed, ultimately causing organelle damage, synaptic dysfunction, and degeneration of neurons in the brain. In double-transgenic APP/PS1 model mice, the autophagy biomarker LC3 (microtubule-associated protein 1 light chain 3) increases with age in neurons, and numerous autophagosomes reportedly accumulate in the cell bodies and axons of nerve cells [16]. However, mice lacking autophagy-related genes (Atg7 and Atag5) in the nervous system exhibit a series of pathological phenomena, including behavioral defects, axonal degeneration, and neuronal loss [17, 18]. Deficiency of lysosomal-associated membrane protein-2 (LAMP-2) in the hypothalamus and hippocampal CA3 region can cause significant inflammation and lysosomal/autophagy disorders, characterized by the accumulation of autophagy vesicles and neuronal degeneration [19]. In VD rats, numerous autophagic vesicle-like structures have been observed in hippocampal CA1 neurons [10], nd evidence of rapid activation of autophagic activity in hypoxic models of cerebral ischemia is growing [20]. Thus, the regulation of autophagy has promising applications as a potential therapeutic target for the treatment of VD.

Intracellular calcium homeostasis is known to be involved in neuronal development and normal physiological functions. As a second messenger, calcium regulates a host of cellular functions such as proliferation, growth, differentiation, and death [21, 22]. Notably, Ca^2+^ plays an essential role as a pro-autophagic signal that can trigger autophagy by phosphorylating serine/threonine-protein kinase ULK1 (unc51-like kinase 1) with an activator site or via the inhibition of rapamycin complex 1 [23]. Furthermore, calcium agonists can promote autophagy by increasing intracellular Ca^2+^ concentration [24]. Responses to Ca^2+^ signals are induced by a family of multifunctional Ca^2+^/calmodulin (CaM)-dependent protein kinases (CaMKs), among which CaMKII is involved in synaptic plasticity and memory formation [25]. Phosphorylated CaMKII reportedly improves learning and memory by promoting synaptic transmission. However, evidence demonstrating abnormally elevated phosphorylated CaMKII protein expression in VD rats has been accumulating [26]. Regulation of the CaMK signaling pathway to control the homeostasis of apoptosis and autophagy can ultimately reduce neurodegenerative processes in the striatum [27]. CaMKII can phosphorylate Beclin 1 directly at Ser90, thereby promoting the ubiquitination of Beclin 1 and activating autophagy in neuroblastoma cells [28]. These data suggest that excessive activation of calcium signaling pathways may induce abnormal changes in autophagy.

Gastrodin (GAS) is one of the bioactive ingredients derived from *Gastrodiae rhizoma* (a Chinese herb named TianMa), extensively employed to prevent and ameliorate diseases of the central nervous system in Chinese medicine [29]. Reportedly, GAS could exert potential effects on VD by targeting multiple pathways as follows: attenuation of amyloid and tau levels, inhibition of autophagy and apoptosis of hippocampal neurons, and alleviation of inflammation [29]. However, whether GAS can ameliorate learning and memory impairment in a rodent model of VD by regulating autophagy via the Ca^2+^/ CaMKII signal remains unclear.

In the present study, we evaluated the effects of GAS on improving autophagic dysfunction in neurons *in vitro* and *in vivo*. GAS ameliorated cobalt chloride (CoCl_2_)-induced autophagic flux inhibition and autophagosome formation in HT22 cells, as well as upstream Ca^2+^ and CaMKII events. Our results suggested that GAS promotes autophagic flux inhibition of Ca^2+^/ CaMKII signaling pathway to improve cognitive impairment in VD. Thus, GAS is a promising drug candidate for ischemia-induced VD.

## RESULTS

### GAS ameliorated cognitive dysfunction in a VD rat model

**Figure 1:**
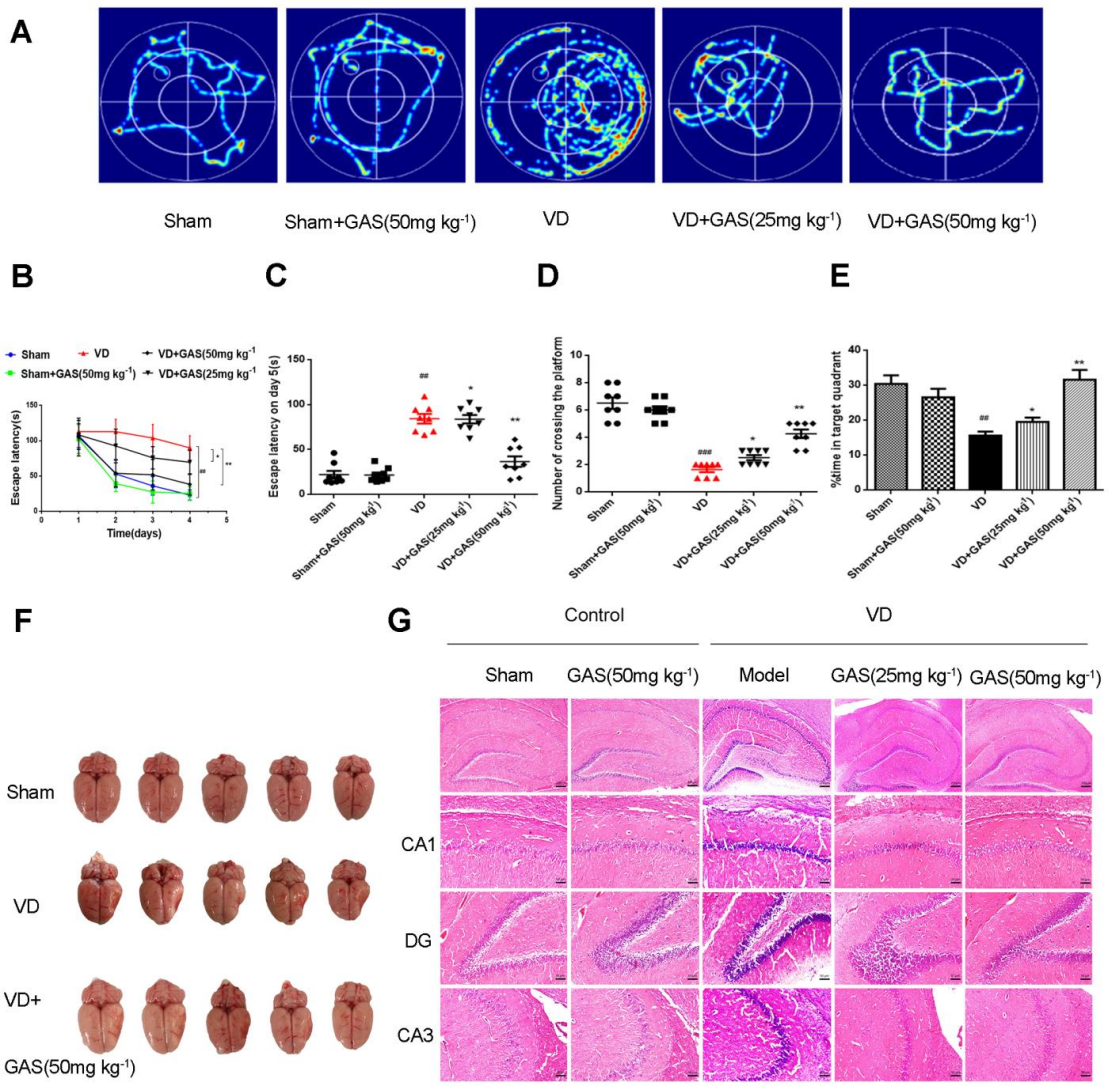
**GAS ameliorated cognitive dysfunction in VD model rats.** (**A**) Typical swimming tracks of VD rat in the Morris water maze test. (**B**) Mean daily escape latency was examined. (**C**) The escape latency of VD model rats is significantly longer than the sham group. After 8 weeks of GAS treatment, the escape latency is significantly shortened in the probe trial. (**D**) The number crossings in the target quadrant in the probe trial. (**E**) Percentage of time spent in the target quadrant in the probe trial. (**F**) Representative photographs of the dissected brain. (**G**) Observation of morphological changes in hippocampal neurons by H&E staining (×50, ×200. Scale bars: 200 μm or 50 μm. Data are presented as the mean ± standard error of the mean (SEM). ^##^*P*< 0.01 versus sham, ^*^P< 0.05, ^**^P< 0.01 versus model. GAS, gastrodin; VD, vascular dementia; H&E, hematoxylin-eosin.

Chronic cerebral hypoperfusion is known to worsen cognitive impairment in memory and learning, resulting in the deterioration of dementia; it is also a pathogenic factor of neurodegenerative diseases [6]. On performing behavioral experiments using the Morris water maze (MWM), our results revealed that permanent middle cerebral artery occlusion (pMCAO) causes learning and memory impairment in rats, whereas GAS significantly improved the assessed parameters. Herein, we reproduced the VD model using the Zea Longa method and determined its success with the Zea Longa score, triphenyltetrazolium chloride (TTC) staining (Supplementary Figure 1), and behavioral experiments. Compared with the normal group, no significant change was observed in the cognitive function of animals in the GAS-only group, indicating that GAS does not affect the cognitive function of normal animals. In navigation tests, the escape latency of VD rats was prolonged when compared with sham-operated rats. In contrast, the escape latency of GAS-treated rats was significantly lower than that demonstrated by VD rats (Figure 1A–1C). In the probe trial, GAS treated VD rats spent more time in the target quadrant, crossing it more frequently (Figure 1D, 1E). This indicated that GAS alleviated the reduced learning and memory function observed in VD rats. In VD, pathological changes are mainly secondary to the occlusion of the trunk of the cerebral artery, resulting in a large area of infarction in the ipsilateral, frontal, parietal, and occipital lobes, most commonly documented in the bilateral or left cerebral hemispheres [4]. Our results showed a significant collapse in the temporal and parietal lobes of the telencephalon in VD rats; GAS attenuated this lesion (Figure 1F). In VD rats, hematoxylin and eosin (H&E) staining of hippocampal tissues showed neuronal disturbances, including loss, degeneration, and necrosis, as well as pyknotic nuclei and light coloration of the cytoplasm. Notably, these pathological characteristics were attenuated following GAS administration (Figure 1G).

### GAS reversed the suppression of autophagic flux and hyperphosphorylation of CaMKII in VD rats

**Figure 2:**
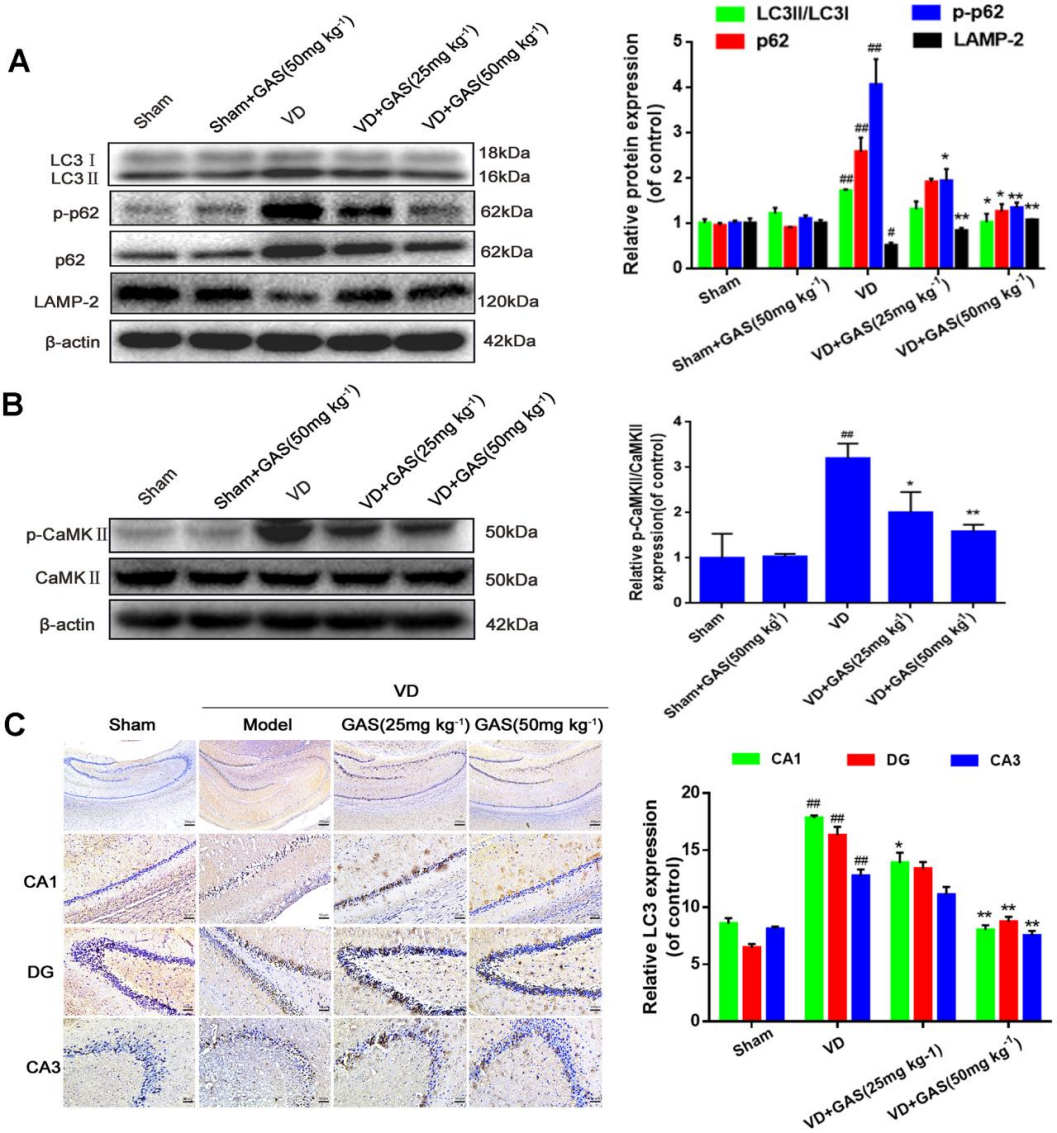
**GAS reversed the suppression of autophagy flux and hyperphosphorylation of CaMKII in VD rats.** (**A**, **B**) The protein extract of hippocampal tissue was analyzed by western blotting for LC3, p62, p-p62 (Thr349), LAMP-2, CaMKII, and p-CaMKIIα (Thr286). Protein levels were quantified and normalized to β-actin. Data are presented as the mean ± standard error of the mean (SEM). ^##^*P*< 0.01 versus sham, ^*^P<0.05, ^**^P< 0.01 versus model. (**C**) Representative images of hippocampal tissue sections immunostained with LC3 antibodies (×50, ×200). Scale bars: 200 μm or 50 μm. LC3, microtubule-associated protein 1 light chain 3; LAMP-2, lysosomal-associated membrane protein-2; CaMKII, Ca^2+^-calmodulin stimulated protein kinase II; p-CaMKIIα, phosphorylated CaMKIIα.

In neurodegenerative diseases, autophagy is abnormal and hence is unable to degrade abnormal proteins in cells, resulting in neurofibrillary tangles and neuronal death [30]. In the present study, we aimed to determine whether GAS could improve the learning and memory impairment observed in VD rats by regulating autophagy. Western blot analysis revealed that LC3 and p62, biomarkers of autophagy induction, increased simultaneously in the hippocampus of VD rats, whereas LAMP-2 expression decreased significantly (Figure 2A). However, the upregulation of LC3 and p62 was suppressed following GAS treatment, which suggested that GAS activated signals downstream of autophagy to promote autophagosome degradation and autophagic flow. Similarly, immunohistochemical results confirmed that GAS decreased LC3 expression when compared with untreated VD rats, especially in the hippocampal CA1 regions (Figure 2C). Autophagy and apoptosis are forms of programmed cell death that play crucial roles in neurodegenerative diseases [31]. Furthermore, GAS may partially play a neuroprotective role by reducing caspase-3 associated cell apoptosis (Supplementary Figure 2). CaMKII, which belongs to the CaMK family, is a serine/threonine-protein kinase that serves as a pivotal calcium signal molecule; it is the main mediator of physiologically excitatory glutamate signals [21, 27, 32]. CaMKII is known to play a critical role in the regulation of autophagy [28]. Herein, although the total CaMKIIα protein expression was unchanged in the hippocampus of VD rats, the phospho-CaMKII-α protein was undoubtedly increased. Thus, GAS can reduce the phosphorylation level of CaMKIIα (Figure 2B).

### GAS alleviated CoCl_2_-induced autophagosome accumulation in HT22 cell

Several studies have confirmed that overactivation of autophagy is involved in the pathogenesis of VD [33, 34]. CoCl_2_, a common chemical reagent, is widely regarded as a classical stimulator of hypoxia-ischemic diseases [35]. The MTT assay showed that the survival rate of HT22 cells significantly decreased after exposure to ≥200 μM CoCl_2_. Pre-incubation with GAS (200 μM) for 1 h followed by exposure to 200 μM CoCl_2_ for 24 h significantly increased the cell survival rate (Figures 3A, 3B).

**Figure 3:**
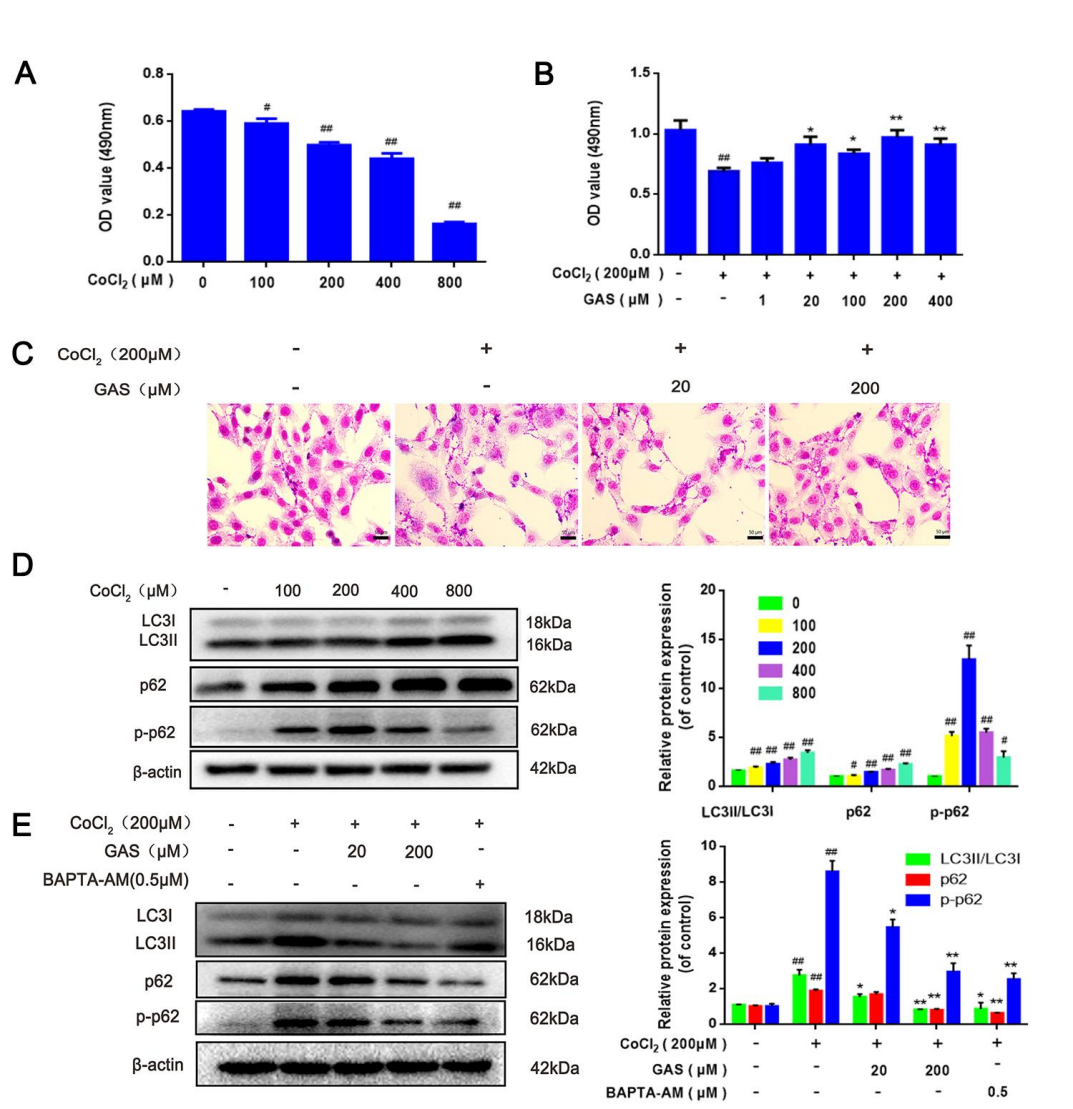
**GAS alleviated CoCl_2_-induced autophagosome accumulation in HT22 cells.** (**A**) HT22 was incubated with different concentrations of CoCl_2_ for 24 h, and cell viability was detected using the MTT assay (n=6). (**B**) Cells were incubated with different concentrations of GAS for 1 h and then treated with CoCl_2_ (200 μM) for 24 h. Cell viability was assessed via the MTT assay (n=6). (**C**) Representative Giemsa staining of HT22 cells (magnification, 200×; n = 3) (**D**) Immunoblots showing levels of LC3, p62, p-p62 (Thr349)in HT22 cells treated with various concentrations of CoCl_2_ (0, 100, 200, 400 and 800 μM) for 24 h. β-actin was used as the loading control. (**E**) HT22 cells were pretreated with GAS and BAPT-AM (0.5μM) for 1 h and then exposed to CoCl_2_ (200μM) for 24 h. Expression of LC3, p62, and p-p62 (Thr349) was detected by immunoblotting. β-actin was used as the loading control.

**Figure 3:**
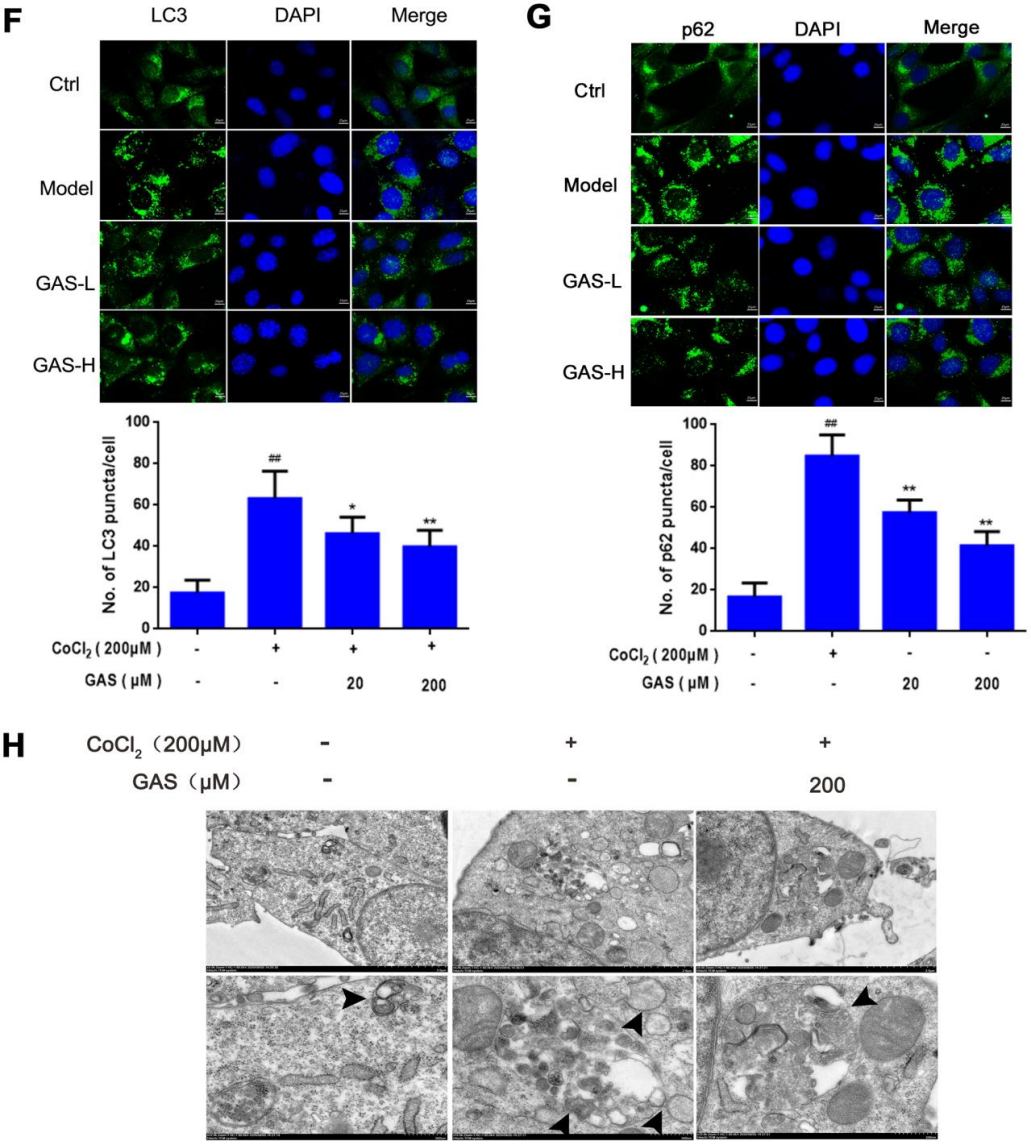
**GAS alleviated CoCl_2_-induced autophagosome accumulation in HT22 cells.** (**F**) Immunofluorescence analysis revealing the modulation of LC3 in HT22 cells with or without GAS for 24 h (n = 3). Autophagosomes were visualized (green puncta) using a Leica DMIRB at 800× magnification. In each independent experiment, 5 visual field cells were randomly selected and quantified, and are expressed as mean ± standard error of the mean (SEM). Immunofluorescence staining of p62 in HT22 cells. (**G**) Immunofluorescence analysis of p62 in HT22 cells, with or without GAS treatment for 24 h (n = 3). (**H**) Representative transmission electron microscopy images in HT22 cells. Arrows denote autophagosomes. Data are presented as the mean ± SEM. ^##^*P*< 0.01 versus control, ^*^P< 0.05 versus CoCl_2_. GAS, gastrodin; CoCl_2_, cobalt chloride; LC3, microtubule-associated protein 1 light chain 3.

To further explore the role of CoCl_2_ in autophagy, HT22 cells were exposed to different concentrations of CoCl_2_ for 24 h. The expression levels of LC3-II significantly increased in a dose-dependent manner, with no significant change in Beclin-1 expression (Figure 3D and Supplementary Figure 3A), which may be attributed to elevated autophagosome formation or suppressed autophagy degradation. In addition to LC3, the level of p62 was significantly upregulated (Figure 3D); p62 is an autophagy substrate that can attach to LC3 and ubiquitinated substrates, which then integrate into autophagosomes to be degraded in autophagolysosomes [36] A considerable amount of aggregated p62 is phosphorylated at a particular amino acid site under the action of protein kinase, finally entering into autophagy lysosomes to complete the degradation of the ubiquitinated substrate [37]. CoCl_2_ exposure significantly enhanced the phosphorylation level of p62 when compared with the control group (Figure 3D). This result suggested that the increase in autophagic vacuoles may be attributed to the inhibition of degradation. Western blotting results revealed that protein expression levels of LC3, p62, and p-p62 were significantly reduced after GAS administration (Figure 3E), indicating that GAS may reduce the accumulation of autophagosomes by decreasing the formation of autophagic vacuoles or promoting autophagic flux. To further validate our results, immunofluorescence of LC3 and p62 proteins was performed using commercial kits. After exposing HT22 cells to CoCl_2_, the number of LC3 and p62 puncta significantly increased. Pre-incubation with GAS significantly reversed these CoCl_2_-mediated changes in autophagy-marker levels (Figure 3F, 3G). Furthermore, GAS inhibited the CoCl_2_-induced excessive accumulation of autophagosomes, as presented in transmission electron microscopy images (Figure 3H).

### GAS ameliorated CoCl_2_-induced autophagic flux inhibition in HT22 cells

**Figure 4:**
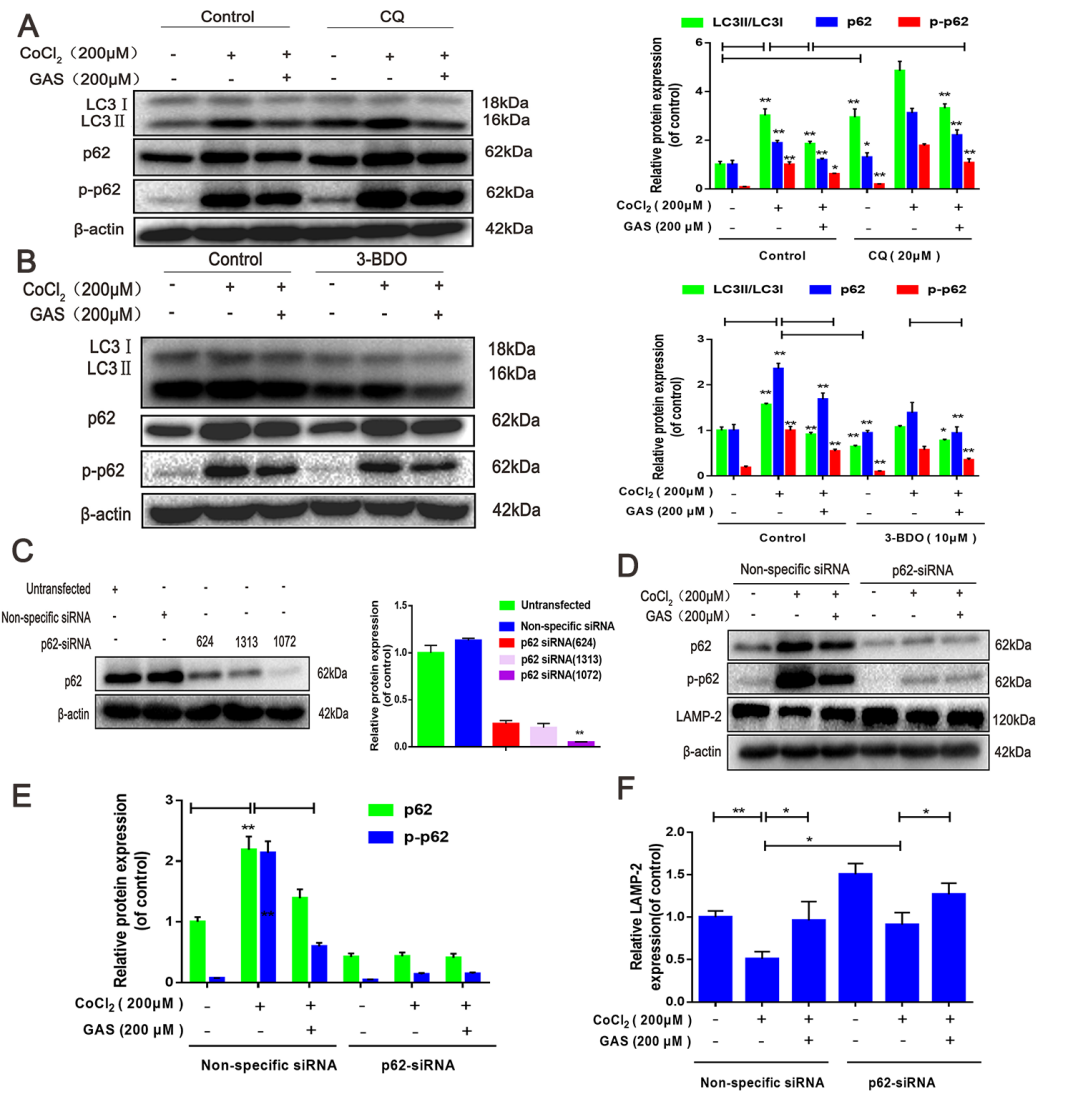
**GAS ameliorated CoCl_2_-induced autophagic flux inhibition in HT22 cells.** (**A**) Levels of LC3, p62, and p-p62 (Ser349) in HT22 cells treated with CQ were assessed, with or without GAS (200 μM) for 24 h (n = 3). (**B**) Levels of LC3, p62, and phosphorylated p62 (Ser349) in HT22 cells treated with 3-BDO were assessed with or without GAS (200 μM) for 24 h (n = 3). (**C**) Detection of p62 siRNA transfection efficiency by western blotting. (**D**–**F**) Levels of LAMP-2, p62, and phosphorylated p62 (Ser349) in HT22 cells transfected with nonspecific or p62 siRNA were evaluated with or without GAS (200 μM) for 24 h (n = 3). The experimental results were normalized to β-actin levels and are shown as fold changes relative to control cells. Data are presented as the mean ± standard error of the mean (SEM) from three independent experiments. ^##^*P*< 0.01 versus control, ^*^P< 0.05 versus CoCl_2_. GAS, gastrodin; CoCl_2_, cobalt chloride; LC3, microtubule-associated protein 1 light chain 3; CQ, chloroquine.

Autophagic flux is a dynamic process, which occurs as a series of continuous steps in cells [38]; hus, an obstacle in any step hinders the completion of biological function. Herein, the effect of GAS on autophagic flux was evaluated in combination with the lysosomal inhibitor chloroquine (CQ). In HT22 cells, treatment with CQ alone induced an increase in LC3, p62, and p- p62 levels. Moreover, CoCl_2_-induced accumulation of LC3-II, p62, and p-p62 was further increased following CQ treatment. Pre-incubation with GAS alleviated the accumulation of LC3-II and p62 in CQ and CoCl_2_-induced suppression of autophagy flow (Figure 4A). These results suggested that GAS alleviated CoCl_2_-induced autophagic flux inhibition, as well as the formation of autophagosomes, in HT22 cells. These GAS effects were further confirmed by examining the impact of the early-stage autophagy inhibitor, 3-benzyl-5-([2-nitrophenoxy] methyl)-dihydrofuran-2(3H)-one (3BDO), on CoCl_2_-induced LC3-II and p62 accumulation in HT22 cells. As expected, 3-BDO alone inhibited the increase in LC3-II, p62, and p-p62 induced by CoCl_2_, with further downregulation observed when GAS was combined with 3-BDO (Figure 4B).

The fusion of autophagosomes and lysosomes is key to the degradation of autophagosomes and their contents, and this process is an important downstream event of autophagic flux [38]. p62 siRNA can improve autophagy by reducing the accumulation of p62 aggregation and promoting autophagic flux [39]. Thus, p62 knockdown by siRNA suppressed the upstream signals of autophagic flux. p62 expression was assessed in HT22 cells transfected with three different p62 siRNAs: siRNA (624), siRNA (1313), and siRNA(1072). Experimental results showed that siRNA (1072) reduced p62 expression, and hence, siRNA (1072) was used in subsequent experiments (Figure 4C). CoCl_2_ significantly reduced LAMP-2 protein expression in HT22 cells, indicating some interference in autophagosome-lysosome fusion. In contrast, GAS or p62-siRNA alone significantly increased LAMP-2 protein expression, whereas GAS combined with p62-siRNA further increased the expression of LAMP-2 (Figure 4D–4F). Collectively, these results suggested that GAS reduced the accumulation of p62 and LC3 aggregation via the autophagy-lysosome pathway to improve autophagic dysfunction.

### GAS alleviated CoCl_2_-induced intracellular Ca^2+^ abundance and CaMKII activation

**Figure 5:**
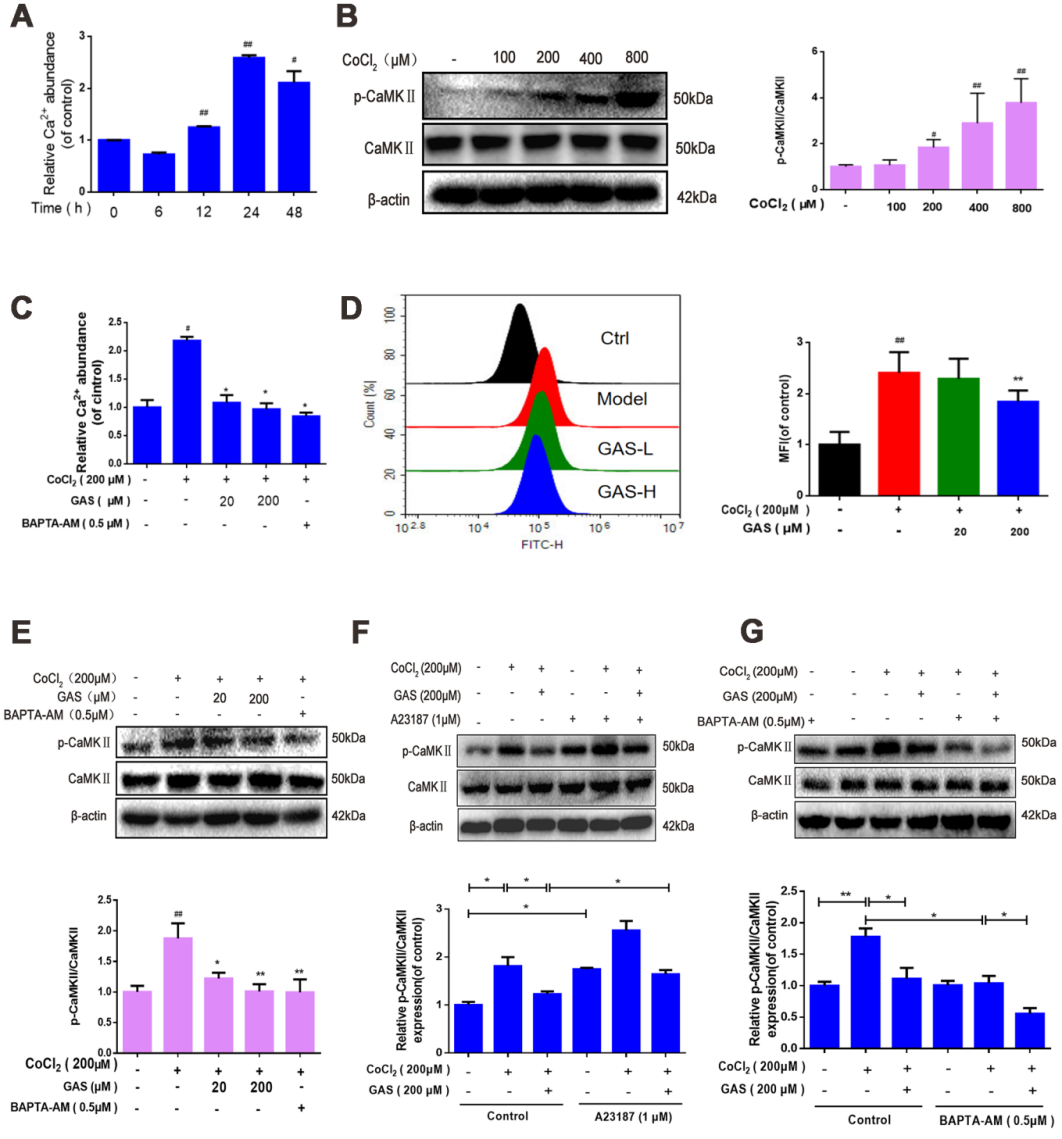
**GAS alleviated CoCl_2_-induced intracellular Ca^2+^ abundance and CaMKII activation.** (**A**) After HT22 cells were treated with CoCl_2_ for different time points, the Ca^2+^ content was detected using a commercial calcium quantitative kit. (**B**) After HT22 cells were treated with different doses of CoCl_2_ for 24 h, phosphorylated CaMKII α increases in a dose-dependent manner. β-actin was used as a loading control. (**C**) HT22 cells were pretreated with GAS and BAPT-AM (0.5 μM) for 1 h and then plated with CoCl_2_ (200 μM) for 24 h; the Ca^2+^ content was detected using a commercial calcium quantitative kit. (**D**) HT22 cells were pretreated with GAS for 1 h and exposed to CoCl_2_ (200 μM) for 24 h; cytosolic Ca^2+^ levels were measured by flow cytometry. (**E**) GAS, similar to calcium chelator (BAPTA-AM), can reduce the level of phosphorylated CaMKII. (**F**–**G**) Levels of CaMKII and phosphorylated CaMKII (Ser249) in HT22 cells treated with calcium chelator (BAPTA-AM) or calcium ionophore (A23187) were detected with or without GAS treatment (200 μM) for 24 h (n = 3). The experimental results were normalized to β-actin levels and are shown as fold changes relative to control cells. Data are presented as the mean ± standard error of the mean (SEM) from three independent experiments. ^##^*P*< 0.01 versus control, ^*^P< 0.05 versus CoCl_2_. GAS, gastrodin; CoCl_2_, cobalt chloride; CaMKII, Ca^2+^-calmodulin stimulated protein kinase II.

The CaMK family is recognized as a key mediator in living organisms and various pathophysiological processes. CaMKII is activated in the presence of Ca^2+^ and CaM [40]. Activation of the Ca^2+^/CaMKII signaling pathway improves learning and memory impairment induced by hypoperfusion [41]. We further explored the effects of GAS and/or BAPTA/AM or calcium ionophore on the intracellular Ca^2+^ level and CaMKIIα phosphorylation in HT22 cells. Our findings revealed that Ca^2+^ abundance significantly increased following exposure to CoCl_2_ for 12 h, peaking at 24 h (Figure 5A). Furthermore, phospho-CaMKII-α increased in a dose-dependent manner (Figure 5B). The Ca^2+^-sensitive fluorescence indicator, Fluo-4 AM, confirmed that GAS and BAPTA-AM, an intracellular Ca^2+^ chelator, inhibited increased intracellular Ca^2+^ and CaMKIIα phosphorylation (Figure 5C–5E). Interestingly, the combination of GAS with BAPTA/AM attenuated the CoCl_2_-triggered [Ca^2+^]_i_ increase and p-CaMKIIα more potently than GAS or BAPTA-AM alone in HT22 cells (Figure 5F), indicating the inhibitory effect of GAS on the increased CaMKIIα phosphorylation induced by CoCl_2_ depending on the level of [Ca^2+^]_i_. Furthermore, the influence of GAS was confirmed using a calcium ionophore, attenuating the inhibitory effect of GAS on the increased intracellular Ca^2+^ and CaMKIIα phosphorylation induced by CoCl_2_ in HT22 cells (Figure 5G).

### GAS alleviated the CoCl_2_-induced suppression of autophagic flux by lowering [Ca^2+^]_i_-dependent CaMKII phosphorylation in HT22 cells

Ca^2+^ is considered a crucial regulator of autophagy, and calcium signaling is closely related to the occurrence and development of autophagy [42]. Accordingly, we further explored the mechanism through which GAS attenuates CoCl_2_-induced inhibition of autophagic flux by regulating [Ca^2+^]_i_-dependent CaMKII phosphorylation in HT22 cells. Co-treatment with GAS and BAPTA-AM significantly inhibited the CoCl_2_-induced increase in LC3, p62, and p-p62 when compared with GAS or BAPTA-AM alone (Figure 6B). Additionally, western blot and immunofluorescence analyses revealed that the inhibitory effects of GAS on CoCl_2_-induced LC3, p62, and p-p62 upregulation were reduced by calcium ionophore treatment (Figure 6A, 6C). These data indicated that GAS ameliorated CoCl_2_-induced autophagic flux inhibition by reducing the extracellular Ca^2+^ influx. CaMKII is a general integrator of Ca^2+^ signaling [43]. CaMKII can phosphorylate Beclin 1 directly at Ser90, thereby promoting the ubiquitination of Beclin 1 and activating autophagy in neuroblastoma cells [28]. Accordingly, we determined whether GAS suppressed autophagy by modulating Ca^2+^-dependent CaMKII. We then confirmed that GAS improved CoCl_2_-induced autophagic flux dysfunction by regulating CaMKIIα. Treatment with KN93, a CaMKII inhibitor, suppressed the CoCl_2_-induced increase in LC3-II, p62, and p-p62 protein expressions. However, no significant difference was observed when compared with KN93 combined with GAS (Figure 6D). To further corroborate the role of CaMKII in the GAS-mediated regulation of autophagy, CaMKII was knocked down; our findings revealed that CaMKII protein expression was downregulated by approximately 70% in HT22 cells transfected with CaMKII-siRNA (379) when compared with nonspecific siRNA-infected HT22 cells (Figure 6E). Interestingly, knockdown of CaMKII significantly inhibited the increase in LC3, p62, and p-p62 induced by CoCl_2_, but no further downregulation was observed when CaMKII knockdown was combined with GAS (Figure 6F). The fusion of autophagosomes and acid lysosomes is an important downstream event of autophagic flux [30]. Our results showed that CoCl_2_ exposure reduced LAMP-2 expression, suggesting that CoCl_2_ hindered the fusion of autophagosomes and acid lysosomes. GAS significantly reversed the CoCl_2_-mediated changes in lysosomal markers, consistent with the results obtained after CaMKII-siRNA knockout. Additionally, the transfection of CaMKII-siRNA followed by GAS administration further increased LAMP-2 expression (Figure 6K, 6M). Co-localization of LC3 II and LAMP-2 is often used to evaluate the fusion of autophagosomes and lysosomes. CoCl_2_ increased LC3^+^ puncta in HT22 cells and weakened the extent of co-localization between LAMP-2 and LC3. GAS increased LAMP-2 expression and decreased the accumulation of LC3^+^ puncta. However, CaMKII knockdown enhanced the co-localization of LAMP-2 and LC3 promoted by GAS (Figure 6G). Additionally, the two peptides AVLFPTLR and IPTGQEYAAK information of mass spectrometry were extract from the present mass spectrometry by professionals from the data, which showed that the p62 interacting proteins contained CaMKII (Figures 6H), which was confirmed by co-immunoprecipitation assay (Figures 6I–6J). However, GAS reduced the binding of p62 and CaMKIIα (Figure 6I, 6J). Collectively, these findings demonstrated that GAS alleviated the CoCl_2_-induced suppression of autophagic flux by lowering [Ca^2+^]_i_-dependent CaMKII phosphorylation in HT22 cells.

**Figure 6:**
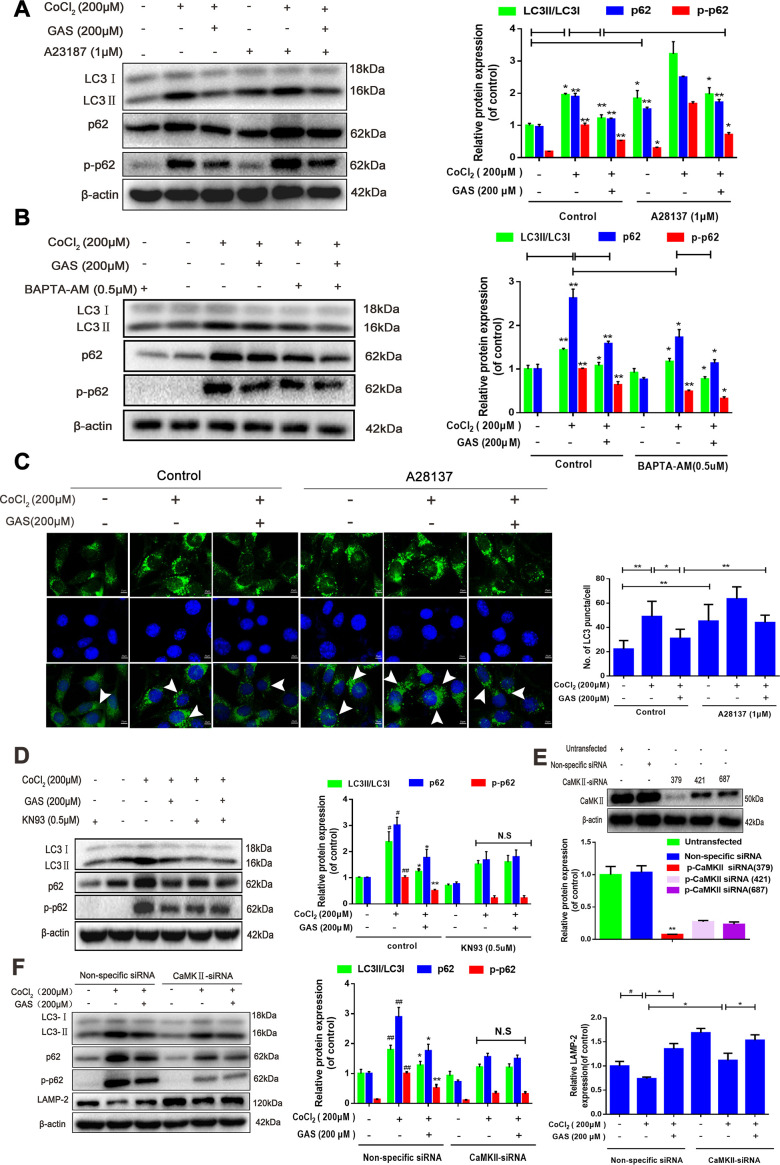
**GAS alleviated the CoCl_2_-induced suppression of autophagic flux by inhibiting [Ca^2+^]_i_-dependent CaMKII phosphorylation in HT22 cells.** (**A**, **B**) Levels of LC3, p62, and phosphorylated p62 (Ser349) in HT22 cells treated with calcium chelator (BAPT-AM) or calcium ionophore (A23187) were assessed with or without GAS treatment (200 μM) for 24 h (n = 3). (**C**) Immunofluorescence analysis revealing modulation of LC3 in HT22 cells treated with calcium ionophore (A23187), with or without GAS (200 μM) for 24 h (n = 3). Autophagosomes were visualized (green puncta) by using a Leica DMIRB at 800× magnification. In each independent experiment, 5 visual field cells were randomly selected and quantified and expressed as mean ± standard error of the mean (SEM). (**D**) Levels of LC3, LAMP-2, p62, and phosphorylated p62 (Ser349) in HT22 cells treated with KN93 were assessed with or without GAS treatment (200 μM) for 24 h (n = 3). (**E**) Detection of CAMKII-siRNA transfection efficiency by western blotting. (**F**) Levels of LC3, LAMP2, p62, and phosphorylated p62 (Ser349) in HT22 cells transfected with nonspecific siRNA or CaMK II-siRNA were evaluated with or without GAS treatment (200 μM) for 24 h (n = 3). The experimental results were normalized to β-actin levels and are shown as fold changes relative to control cells.

**Figure 6:**
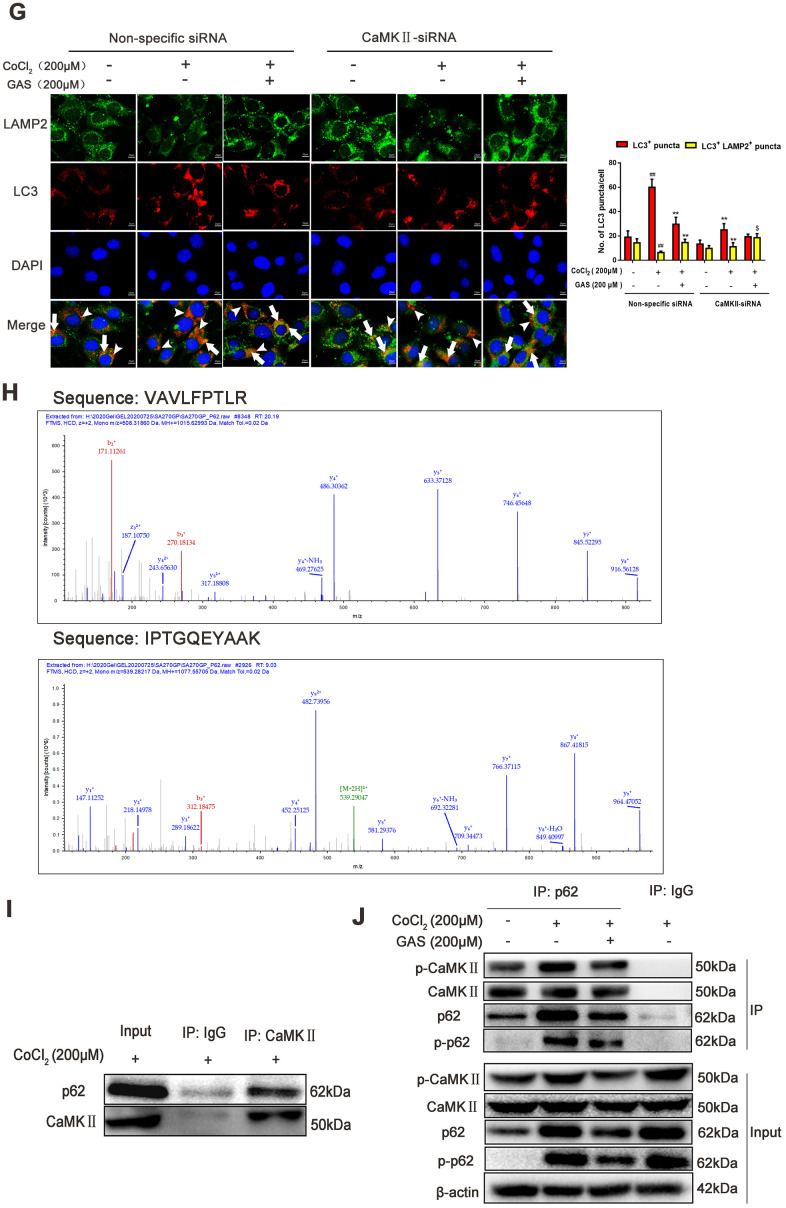
**GAS alleviated the CoCl_2_-induced suppression of autophagic flux by inhibiting [Ca^2+^]_i_-dependent CaMKII phosphorylation in HT22 cells.** (**G**) Coimmunostaining of LC3 with LAMP2 in HT22 cells. LC3^+^ puncta (green puncta) and LC3^+^LAMP2^+^ puncta (yellow puncta) were visualized using a Leica DMIRB at 800× magnification. In each independent experiment, 5 visual field cells were randomly selected and quantified. (**H**–**J**) The interaction between CaMKII and p62 was analyzed using immunoprecipitation and mass spectrometry. Data are presented as the mean ± SEM from three independent experiments. ^##^*P*< 0.01 versus control, ^*^P< 0.05 versus CoCl_2_, ^$^*p*<0.05 versus CoCl_2_ + CaMKII-siRNA. GAS, gastrodin; LC3, microtubule-associated protein 1 light chain 3; LAMP-2, lysosomal-associated membrane protein-2; CaMKII, Ca^2+^-calmodulin stimulated protein kinase II.

## DISCUSSION

The role of autophagy is to prevent the accumulation of abnormal cytoplasmic proteins in neurons, and disrupted autophagy may result in neurodegeneration, characterized by an extensive neuronal loss [17, 44]. Excessive autophagy reduces the survival rate of neurons, and inhibition of autophagy delays the process of neurodegeneration [45], suggesting that drug interventions during autophagy may be a promising novel strategy for dementia treatment. VD is a chronic cerebrovascular syndrome, presenting vascular brain tissue damage as the main pathological manifestation. Learning and memory impairment occurs as neural networks are altered at physiological, molecular, and synaptic levels. However, whether neuronal autophagy occurs in VD remains unclear. Accumulating evidence shows that autophagy is widely activated in the hippocampus of rats presenting VD [46], suggesting that autophagy is involved in the pathogenesis of VD. Furthermore, some studies have reported the existence of copious autophagy vacuoles in the axons of neurons in rats with VD [47, 48], which may reflect the enhancement of autophagy induction, obstruction of late lysosome degradation, or decrease in autophagy initiation rate in autophagy pathways. The current results showed that learning and memory were impaired in the VD modeled rats. Furthermore, excessive autophagosomes were associated with the progression of VD induced by pMCAO, as characterized by a significant increase in LC3 and p62 and a remarkable decrease in LAMP-2 in the hippocampus (Figure 2). These results suggested that hippocampal neurons were damaged when lysosome-autophagosome fusion was hindered, which may be one of the causes for learning and memory impairment in VD rats.

GAS, a phenolic glycoside isolated from the traditional Chinese medicine *G. Rhizoma*, has wide application prospects in the prevention and treatment of VD [29]. Previous data have confirmed that GAS has multiple pharmacological properties such as antioxidation, anti-inflammatory, and anti-apoptotic activities. Liu et al. have reported that GAS exerts a therapeutic effect on bilateral common carotid artery occlusion (BCCAO)-induced VD by targeting the formation of Aβ-related proteins and inhibiting autophagy and apoptosis of hippocampal neurons [49]. Yang et al. have revealed that GAS attenuates the methamphetamine-induced death of SH-SY5Y cells by an anti-autophagy effect [50]. A growing number of studies have shown that autophagy disorders play a key role in most neurodegenerative diseases, and autophagy regulation is considered a potential strategy to treat these diseases [51]. In the present study, we confirmed that GAS alleviated learning and cognitive impairment in VD rats, and reversed the hyperphosphorylation of CaMKIIα and abnormal upregulation of the autophagy biomarker proteins, LC3 and p62, in the hippocampus of VD rats. These results indicate that reducing excessive autophagosome formation and downregulating the phosphorylation level of CaMKIIα could be important molecular mechanisms of GAS in preventing VD. However, increased LC3 and p62 levels indicated that autophagy was promoted in the early stage and inhibited in the later stage (binding to lysosomes) or blocked the degradation of autophagy lysosomes. GAS could improve cognitive impairment in VD rats via multiple mechanisms, and the exact regulatory mechanism associated with excessive autophagy in VD rats warrants further investigation.

Notably, autophagy is the self-protective mechanism of cells, beneficial for their growth and development. However, excessive autophagy may lead to metabolic stress, degradation of cellular components, and cell death [11]. Furthermore, as an important metabolic activity, autophagy plays an important role in maintaining neuronal survival, clearing senescent cells, and misfolded proteins under stress such as ischemia and hypoxia [44, 45]. LC3, p62, and LAMP-2 are known to be frequently used autophagy biomarkers. The amount of accumulated autophagosomes is proportional to the content of LC3-II or the ratio of LC3- /LC3-I, and LC3 reflects the autophagy activity of cells to a certain extent [11, 52]. In the present study, we observed that treatment with CQ alone increased the expression of LC3-II and p62, with further upregulation observed when CQ was combined with CoCl_2_; GAS reduced the upregulation of LC3 and p62 (Figure 4). Meanwhile, the early-stage autophagy inhibitor, 3-BDO, alone inhibited the increased LC3-II and p62 expression induced by CoCl_2_, and further downregulation occurred when GAS was combined with 3-BDO. These results suggested that GAS ameliorated the suppression of autophagic flux and inhibited autophagosome formation. Additionally, autophagy is a highly dynamic and multi-stage process. Considering the changes in autophagy activity, evaluation of autophagic flow, including dynamic changes in autophagosome formation, the fusion of autophagosomes and lysosomes, and substrate degradation is necessary [53]. LAMP-2 protein is the key molecule in the final process of autophagy, mediating the fusion between autophagosomes and lysosomes [54]. Furuta et al. have observed the absence of LAMP-2 induced inflammatory changes and lysosome accumulation within neurons of the central nervous system [55]. Herein, we observed that GAS increased the expression of LAMP-2, as well as the binding between autophagosomes and lysosomes (Figure 2). These results showed that GAS alleviated CoCl_2_-induced autophagic flux inhibition and the formation of autophagosomes by promoting lysosomal acidification and autophagosome-lysosome fusion in HT22 cells.

Intracellular free calcium (Ca^2+^), an intracellular second messenger, plays a complex signal transduction role in the brain, especially in neurodegenerative diseases [41, 56]. Impairment of the intracellular Ca^2+^ regulatory system ultimately results in synaptic dysfunction, damaged plasticity, and neuronal degeneration [56]. The depletion of energy induced by cerebral blood flow in patients with VD results in the accumulation of free calcium in cells [57]. The increase in cytoplasmic Ca^2+^ could enhance the binding of the Ca^2+^-CaM complex to the regulatory domain of CaMKII, increasing CaMKII activity, and activating autophagy [25, 42]. The hippocampus and cerebral cortex are the structural basis of spatial learning, and CaMKII is the molecular basis of spatial learning and memory [58]. CaMKIIα is extremely abundant and almost completely expressed in the brain, accounting for more than 1% of the total protein in regions such as the hippocampus [59]. For a prolonged period, the generation of CaMKII autonomy via automatic phosphorylation on Thr-286 has been considered a prominent feature of CaMKII regulation [60, 61]. CaMKII is known to possess 28 different subtypes, among which the threonine 286 residue controls self-inhibition. When the site is phosphorylated, CaMKII is permanently activated, and the long-term synaptic pathway enhances learning and memory ability [62]. In HT22 cells, we observed that GAS reduced the increase in intracellular calcium and CaMKIIα phosphorylation at Ser286, which were induced by CoCl_2_. The intracellular Ca^2+^ chelator BAPTA-AM caused GAS to inhibit the increase in intracellular Ca^2+^ and CaMKIIα phosphorylation, whereas calcium ionophore (A23187) demonstrated the opposite effect (Figure 5). Our study revealed that the neuroprotective effect of GAS was regulated by the interruption of CaMKII signaling. Under adequate nutrition, the autophagy of most cells is maintained at a low basic level. Conversely, under stress conditions (e.g., accumulation of damaged organelles and Ca^2+^ overload in the cytoplasm), autophagy is activated to maintain cell stability, which is conducive to cell survival [63]. Additionally, Ca^2+^ is considered an important regulator of autophagy [64]. We demonstrated that chelating intracellular Ca^2+^ with BAPTA-AM enhanced the inhibitory effect of GAS on the CoCl_2_-induced upregulation of LC3 and p62, whereas calcium ionophore (A23187) demonstrated the opposite effect. However, GAS treatment did not induce any obvious change in LC3 and p62 levels in HT22 cells transfected with CaMKII-siRNA (Figure 6F). These results strongly support the beneficial role of GAS in preventing CoCl_2_-elevated [Ca^2+^]_i_-dependent CaMKII phosphorylation, thereby improving autophagy-lysosome dysfunction. However, the detailed molecular mechanism of GAS in improving autophagy dysfunction warrants further investigation.

Calcium-related pathways are primarily concentrated in the Ca^2+^-CaMKII-AMPK-mTOR pathway [65, 66], but their effects on autophagy are rarely reported. In the present study, for the first time, we revealed that GAS may improve autophagy dysfunction by reducing the binding of CaMKII to p62. p62 protein is located at the site of autophagosome formation and can bind to the autophagosome localization protein LC3 and ubiquitin protein. Thus, p62 is a recognition receptor for ubiquitin and organelle degradation [36, 37]. Decreased p62 levels result in neuropathological changes, including excessive accumulation of tau and Aβ proteins, and even neuronal apoptosis [67]. Indeed, p62 presents different phosphorylation sites and is in dynamic equilibrium between different phosphorylation states. When the inhibition of autophagy results in the intracellular accumulation of p62, phosphorylation of p62 occurs at specific amino acid sites under the action of protease (main phosphorylation sites are Ser403 and Ser351) and directly activates other signaling pathways, including nuclear factor-κB (NF-κB) signaling, Nrf2 activation, and apoptosis [68–70]. Herein, we observed that KN93 (a CaMKII inhibitor) or CaMKII knockdown did not further decrease the total p62 and phosphorylated p62 (Ser349) levels after incubation with GAS (Figure 6D–6F). Additionally, a co-immunoprecipitation assay and mass spectrometry showed that GAS reduced the combination of p62 and CaMKII (Figure 6H–6J).

**Figure 7:**
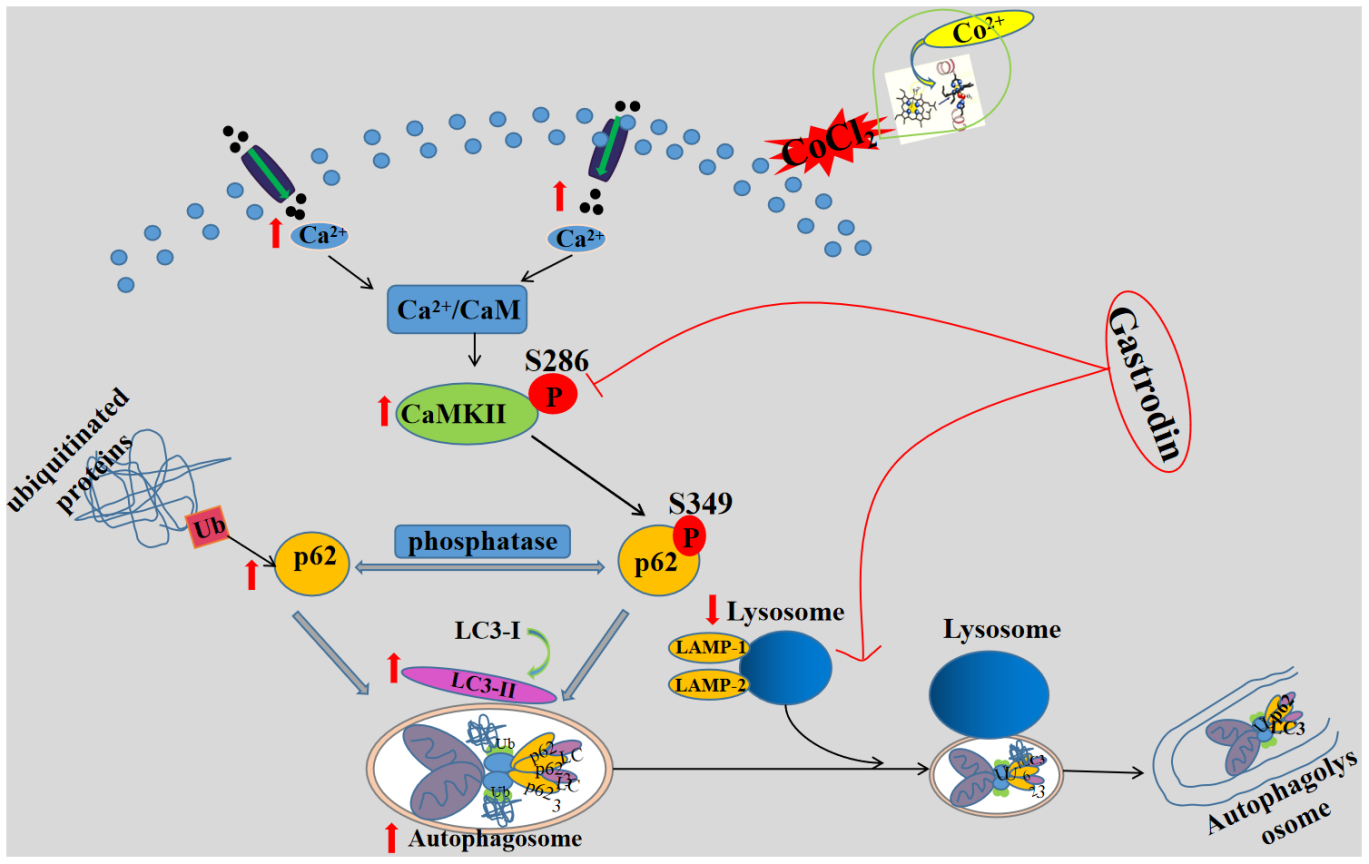
**A schematic diagram of the possible mechanism of gastrodin improving cognitive impairment in vascular dementia rats by promoting autophagy flux through inhibiting Ca^2+^/CaMKII signal pathway.** GAS improves cognitive dysfunction in a VD rat model. GAS ameliorates the CoCl2-induced suppression of autophagic flux by lowering [Ca2+]i-dependent CaMKII phosphorylation in HT22 cells, reduces apoptosis in HT22 cells.

To the best of our knowledge, this report is the first to confirm that GAS significantly reversed cognitive deficits in VD rats and alleviated the accumulation of p62 and LC3 aggregation via the autophagy-lysosome pathway, thus improving autophagy dysfunction. The mechanism of GAS may involve the attenuation of autophagic flux dysfunction by inhibiting the Ca^2+^/CaMKII signaling pathway to ameliorate cognitive impairment in VD (Figure 7).

## MATERIALS AND METHODS

### Reagents and antibodies

GAS (SMB00313), CoCl_2_ (232696), and calcium ionophore (A23187) were purchased from Sigma-Aldrich (St Louis, MO, USA). GAS and CoCl_2_ were dissolved in phosphate-buffered solution to prepare the original solution of 100 mM, which was stored at -20° C. CQ diphosphate (HY-17589) and 3-BDO (HY-U00434) were purchased from MedChemExpress. Fluo-4AM (S1060) was obtained from Beyotime Biotechnology, Inc. (Nanjing, China). Primary antibodies against β-actin (66009-1-Ig, 1:10 000), LC3 (14600-1-AP, 1:1000), p62 (18420-1-AP, 1:1000), p62(66184-1-lg), LAMP-2 (66301-1-lg, 1:1000), and CaMKIIα (11533-1-AP, 66843-1-Ig) were purchased from Proteintech (Wuhan, China). Rabbit anti-p-p62 (Thr349) (Ab211324) was purchased from Abcam (Cambridge, MA, USA). Primary antibodies against CaMKIIα (#4436, 1:1000) and anti-p-CaMKIIα (Thr286) (#12716, 1:1000) were obtained from Cell Signaling Technology (Danvers, MA, USA). Goat anti-rabbit IgG (H&L)-HRP (BS13278, 1:10000) and goat anti-mouse IgG (H&L)-HRP (BS12478) were obtained from Bioworld. Goat PAb to Rb IgG Alexa Fluor®488 (Ab150077, 1:200) and goat PAb to MS IgG Alexa Fluor®647 (Ab150115) were purchased from Abcam. A fluorescein isothiocyanate-conjugated goat anti-rabbit secondary antibody (A0516, 1:200) was purchased from Beyotime Biotechnology.

### Animals and treatments

Healthy adult male Sprague–Dawley rats (SPF grade, Certificate No. SCXK2018-0001, Grant No. 1800157), weighing 260 ± 20 g, were obtained with approval from the Guizhou Medical University Experimental Animal Center. All animals were maintained in pathogen-free rooms with temperature and humidity controlled at 22-26° C and 40%–70%, respectively, under a 12 h:12 h light/dark cycle. All procedures involving experimental animals were approved by the Animal Ethics Committee of Guizhou Medical University.

Focal cerebral ischemia injury was induced by modifying a previous MCAO [71]. In a typical procedure, the left common carotid artery (CCA), external carotid artery (ECA), and internal carotid artery (ICA) were separated with bluntness, and then the ECA was ligated. The suture (0.26 mm; Beijing Xinong BioTechnologies Co., Ltd., China) from the CCA to the ICA eventually occluded the middle cerebral artery. Sham-operated rats received all surgical protocols without the suture inserted. Zea Longa scoring [71] was employed to evaluate neurological deficits after 12 h of ischemia. Rats with scores of 1 and 2 were selected as model rats. TTC staining was used to measure infarct volumes after 24 h of ischemia. Rats were sacrificed after CO_2_ euthanasia, and the brain tissue was quickly removed and cut into 2 mm coronal sections. These sections were incubated in 0.2% TTC at 37° C for 30 min, and cerebral infarction was observed. The samples were photographed with a digital camera at designated time points. The model rats were randomly grouped into five groups (n = 8 per group) as follows: sham, sham+GAS (50 mg kg^-1^), VD, VD+GAS (25 mg kg^-1^), and VD+GAS (50 mg kg^-1^). All animals were administered GAS or distilled water via oral gavage, daily for 8 weeks on day 7 after surgery.

### Morris water maze test (MWM)

Spatial learning and memory were assessed using the MWM paradigm. The water maze consisted of a large circular pool (120 cm in diameter, 50 cm in height) filled with white non-toxic powder. The pool was divided into four equal quadrants, and a hidden circular, platform 20 cm in diameter, was located in the center of the target quadrant. The system automatically recorded the rat’s trajectory when placed in the water from the pool edge. The rats that failed to locate the platform within 120 s were directed by the investigator to the platform and allowed to rest on it for at least 20 s. On day 5, the space exploration experiment was performed, and the system automatically recorded the movement trajectory of the rat within 120 s.

### Hippocampal morphology observation

Brain tissue was fixed in 4% paraformaldehyde for 24 h, embedded in paraffin, and sectioned to analyze hippocampal morphology by H&E staining. Protein expression of LC3 was evaluated by immunohistochemical methods. Brain tissue sections were deparaffinized, and antigen retrieval was achieved by microwave heating in 0.01 M citrate buffer for 20 min. The sections were sequentially incubated with 3% H_2_O_2_ for 15 min and blocked with goat serum for 30 min. Then, the primary antibody LC3 was added dropwise and incubated overnight at 4° C. Next, sections were incubated with the secondary antibody for 30 min, followed by the addition of horseradish-labeled streptavidin in the working solution, and further incubation for 20 min. Finally, the samples were stained with DAB (ZLI-9018, Zhongshan Golden Bridge Biotechnology, Beijing, China) substrate, counterstained with hematoxylin, and photographed using a light microscope (DMi8, Leica, Germany). The immunofluorescence-positive area of hippocampal tissue was assessed using ImageJ image analysis software (National Institutes of Health, Bethesda, MD, USA).

### Cell lines and cell culture

HT22 cells were purchased from Shanghai Zhongqiaoxinzhou Biotech (Shanghai, China; passage number: ZQ0476) and maintained in Dulbecco’s modified Eagle medium (Gibco, Thermo Fisher Waltham, CA, USA) supplemented with 10% fetal bovine serum (Gibco), streptomycin (100 μg/mL; Gibco), and penicillin (100 U/mL; Gibco) before incubation at 5% CO_2_ and 37° C.

### MTT assay

HT22 cell viability was assessed using the MTT assay. In brief, cells were seeded onto 96-well plates and cultured until 70% confluency. The cells were pre-incubated with the final concentrations of GAS (200 μM) for 1 h and then exposed to CoCl_2_ (200 μM) for 24 h. After discarding the medium, 200 μL of 0.5 mg/mL MTT solution was added to each well and incubated for 3 h. Then, the supernatant was removed and 150 μL of dimethylsulfoxide was added, followed by thorough mixing. Optical density (OD) values were measured at 490 nm using a microplate reader (Thermo Fisher Scientific, Waltham, MA, USA). The cell survival rate (%) was calculated using the following equation:

cell viability inhibition(%) = [(OD_Control_ − OD_Treated_)/OD_Control)_] × 100.

### Western blot analysis

Total protein samples from tissues or cells were extracted using a lysis buffer (R0010, Solarbio) containing a phosphatase inhibitor cocktail (P1260, Solarbio) and a protease inhibitor cocktail (P0100, Solarbio). The protein concentration was quantified using a bicinchoninic acid protein assay kit (PC0020, Solarbio) and detected with a microplate spectrophotometer (Thermo Fisher Scientific). Approximately 30 μg of protein samples were fractionated on a 10% or 12% SDS-PAGE gel. After electrophoretic transfer to a polyvinylidene fluoride membrane (Millipore, Bedford, MA, USA), the membrane was blocked with 5% bovine serum albumin (A8020, Solarbio) for 1.5 h and subsequently probed overnight with the appropriate antibodies at 4° C.

### Measurement of intracellular Ca^2+^ level

Intracellular Ca^2+^ levels were quantified using a Ca^2+^ quantification kit (Abcam, ab112115) following the standard manufacturer’s protocol. Fluorescence was determined using a microplate spectrophotometer at Ex/Em = 540/590 nm (Varioskan LUX, Thermo). Additionally, cytosolic Ca^2+^ levels were measured by flow cytometric estimation of Fluo-4 AM. The cells were collected and loaded with 5 μM Fluo-4 (Beyotime, ab145254) for 30 min at 37° C, and then resuspended in 500 μL of phosphate-buffered saline. Fluorescence signals were recorded with a flow cytometer at Ex/Em =488/525 nm and analyzed with NovoExpress software (NovoCyte, ACEA Biosciences, San Diego, CA, USA).

### Gene silencing

CaMKII-siRNA (forward, 5-GCGGAGGAAACAAGAAGAATT-3; reverse, 5-UUCUUCUUGUUUCCUCCGCTT-GTC-3) and p62 siRNA (forward, 5-GACGAUGACUGGACACAUUTT-3; reverse, 5-AAUGUGUCCAGUCAUCGUCTT-3) were constructed by GenePharma (Shanghai, China). According to the protocol instructions, Lipofectamine 2000 reagent (Invitrogen, Carlsbad, CA, USA) was used to transfect siRNA.

### Immunofluorescence microscopy

HT22 cells were seeded onto coverslips from six-well plates treated in accordance with the experimental design. At designated time points, the cells were treated as follows: fixed with 4% paraformaldehyde for 12 min, permeabilized for 10 min using 0.2% Triton X-100, and covered with goat serum (SL038, Solarbio). Then, the cells were incubated with LC3 (1:200), P62 (1:50), and LAMP-2 (1:200) overnight at 4° C, followed by incubation with secondary antibodies for 1 h, and labeling with DAPI (BD5010, Bioworld) for 40 min. Finally, the images were observed with a DMi8 fluorescence microscope and Leica X software at 800× magnification (DMi8, Leica, Germany).

### Co-immunoprecipitation and mass spectrometry

Total lysates from HT22 cells were immunoprecipitated with p62 antibody or the corresponding IgG control overnight at 4° C and then precipitated with protein A/G plus agarose overnight at 4° C (Sangon Biotech, Shanghai, China). Immunoprecipitated proteins were separated by SDS-PAGE, followed by western blotting with the corresponding antibodies or Coomassie blue staining, subjecting them to LC-MS/MS for sequencing and data analysis.

### Statistical analysis

All data are expressed as the mean ± standard error of the mean (SEM) using GraphPad Prism®5.0 (La Jolla, CA, USA). The experimental data were obtained from three or more independent experiments, and differences between groups were analyzed by one-way ANOVA followed by post hoc Tukey multiple comparisons. Differences between the two experimental groups were compared using the Student’s *t*-test. A *P*-value of < 0.05 indicated that the difference was statistically significant.

## Supplementary Material

Supplementary Figures
