# Supplementary material for: Gastrodin ameliorates learning and memory impairment in rats with vascular dementia by promoting autophagy flux via inhibition of the Ca2+/CaMKII signal pathway
Source: Aging (Albany NY). 2021 Mar 10;13(7):9542–65. doi: 10.18632/aging.202667 (PMC8064221; doi:10.18632/aging.202667)
Supplement: Supplementary Figures [file aging-13-202667-s001.pdf]

SUPPLEMENTARY FIGURES

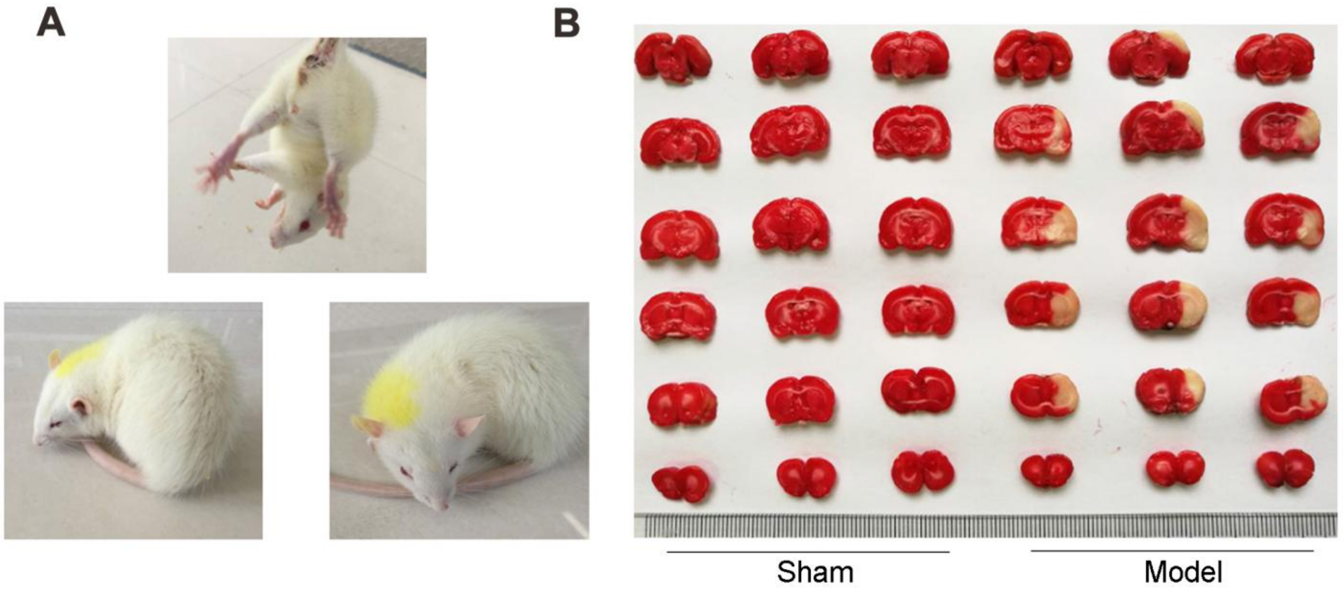

Supplementary Figure 1. Establishment of a rat model of vascular dementia. (**A**) Representative images of "Longa scoring". (**B**) Tetrazolium Chloride (TTC) staining in rat brain tissue.

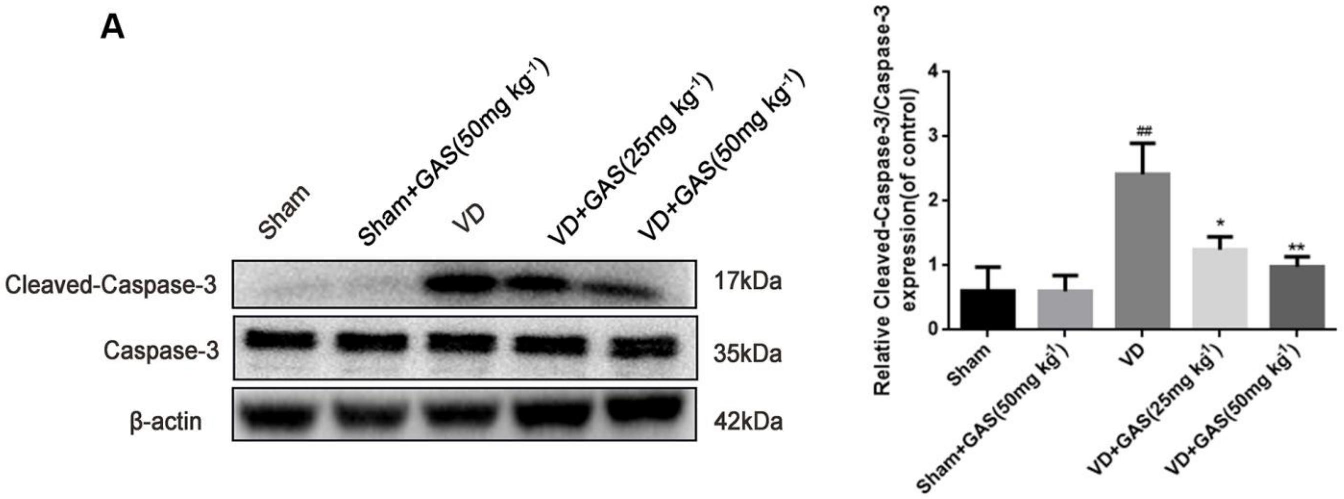

Supplementary Figure 2. Effects of GAS on the protein expression of Caspase-3 in the hippocampus tissues of rats. (**A**) The protein extract of hippocampal tissue was analyzed by Western blotting with Cleaved-Caspase-3 and Caspase-3 (left). Protein levels were quantified and normalized to β-actin (right). Data represent the mean ± SEM. $^{##}P< 0.01$ versus sham, $^{*}P<0.05$, $^{**}P< 0.01$ versus model.

A

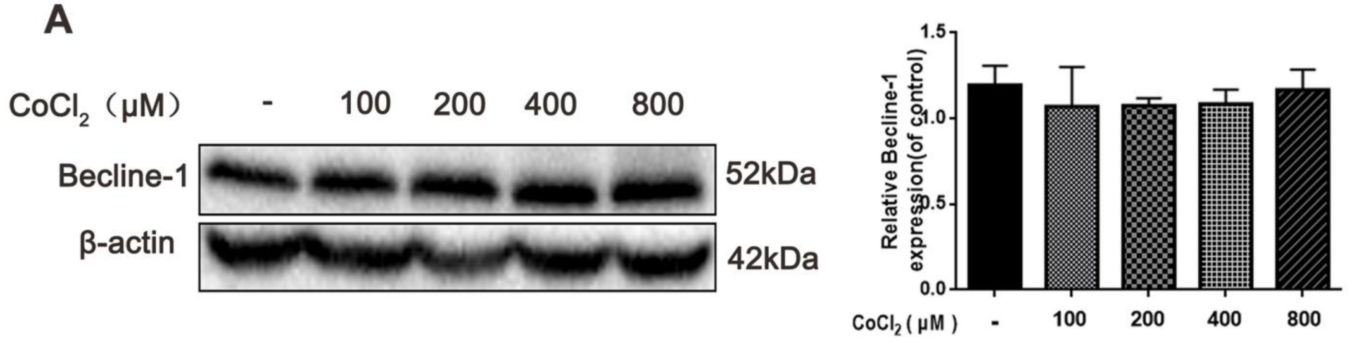

Supplementary Figure 3. Effects of $CoCl_2$ on the expression of autophagy-related proteins in HT22 cells. (**A**) Immunoblots showed levels of Becline-1 in HT22 cells treated with various concentrations of $CoCl_2$ (0, 100, 200, 400 and 800 μM) for 24 h. β-actin was used as loading control. Data represent the mean ± SEM. $^{\#\#}P< 0.01$ versus control.
